# Discovering Health Benefits of Phytochemicals with Integrated Analysis of the Molecular Network, Chemical Properties and Ethnopharmacological Evidence

**DOI:** 10.3390/nu10081042

**Published:** 2018-08-08

**Authors:** Sunyong Yoo, Kwansoo Kim, Hojung Nam, Doheon Lee

**Affiliations:** 1 Department of Bio and Brain Engineering, Korea Advanced Institute of Science and Technology (KAIST), Daejeon 34141, Korea; syyoo@kaist.ac.kr (S.Y.); only826kaist@kaist.ac.kr (K.K.); 2 Bio-Synergy Research Center, Daejeon 34141, Korea; 3 School of Electrical Engineering and Computer Science, Gwangju Institute of Science and Technology (GIST), Gwangju 61005, Korea

**Keywords:** phytochemical, health benefits, network medicine, molecular analysis, ethnopharmacology, herbal medicine, chemical property

## Abstract

Identifying the health benefits of phytochemicals is an essential step in drug and functional food development. While many in vitro screening methods have been developed to identify the health effects of phytochemicals, there is still room for improvement because of high cost and low productivity. Therefore, researchers have alternatively proposed in silico methods, primarily based on three types of approaches; utilizing molecular, chemical or ethnopharmacological information. Although each approach has its own strength in analyzing the characteristics of phytochemicals, previous studies have not considered them all together. Here, we apply an integrated in silico analysis to identify the potential health benefits of phytochemicals based on molecular analysis and chemical properties as well as ethnopharmacological evidence. From the molecular analysis, we found an average of 415.6 health effects for 591 phytochemicals. We further investigated ethnopharmacological evidence of phytochemicals and found that on average 129.1 (31%) of the predicted health effects had ethnopharmacological evidence. Lastly, we investigated chemical properties to confirm whether they are orally bio-available, drug available or effective on certain tissues. The evaluation results indicate that the health effects can be predicted more accurately by cooperatively considering the molecular analysis, chemical properties and ethnopharmacological evidence.

## 1. Introduction

Plants provide not only essential nutrients needed for life, but also other bioactive phytochemicals that contribute to health promotion and disease prevention. While the macro- and micronutrients in plants were long thought to be one of the essential components for human health, phytochemicals have recently emerged as modulators of key cellular signaling pathways [1,2]. Phytochemicals, often called secondary metabolites, are non-nutritive chemical compounds produced by plants via several chemical pathways. Recent studies have demonstrated that a large number of phytochemicals can be beneficial to the function of human cells [3,4,5]. With several studies indicating the effects of phytochemical-rich foods on health, it is strongly suggested that ingesting these phytochemicals can help to improve health [6,7,8]. Based on such evidence, many researchers have previously conducted studies to investigate the roles of phytochemicals in health improvements.

Despite the efforts, studies on the precise roles of phytochemicals were faced with various limitations. To begin with, most of the studies were performed through in vitro assessment [9,10,11]. For example, in vitro screening methods were used to confirm biological activities of extracted phytochemicals. However, large-scale experiments are required for a large number of considered phytochemicals and potential health effects, a process which is costly and yet very unproductive. Therefore, in silico approaches, mostly based on molecular or ethnopharmacological information, have been proposed to identify the potential health effects of phytochemicals from numerous candidates. Molecular-based approaches focus on the similarity between phytochemicals and approved drugs, such as the molecular structure, mechanisms of the molecular network or target protein similarity, to predict potential effects of phytochemicals [12,13,14]. However, these approaches are designed to predict the specific effect of phytochemicals on specific phenotypes, or vice versa. Therefore, it is difficult to analyze the systemic health effects on the human body. Alternatively, some ethnopharmacological knowledge-based approaches have been developed [15,16,17,18]. These studies focused only on the ethnopharmacological information as a preliminary tool to select plants or phytochemicals for a certain disease, followed by molecular analysis or in vitro assessment. Although this process is useful to filter out phytochemicals from a large number of candidates, the productivity is still low because plants contain hundreds of phytochemicals. Moreover, it is difficult to find plants that are highly related to a particular health effect, since effect terms are closely related to each other [19,20]. For example, when extracting plants associated with urination, we need to consider the phenotypes associated with urination, such as dysuria, urethral stone, and urinary tract abnormalities, to achieve more relevant results. These problems make it difficult to perform large-scale analysis of phytochemicals.

In this study, we apply an integrated in silico analysis to identify the potential health benefits of phytochemicals. Our previous study demonstrated that phenotypic effects of drugs can be identified by investigating the propagated drug effects from a molecular network, and mapping these results to phenotypes [21]. Therefore, we inferred the potential health effects of phytochemicals by adapting our previous method. However, this approach does not provide detailed information about the effects, such as whether they are beneficial, harmful, or associated. To solve this problem, we utilized the ethnopharmacological evidence of plants. Our underlying hypothesis is that if a predicted health effect of a certain phytochemical agrees with the ethnopharmacological use of a large number of plants which contain the phytochemical, then we can reasonably argue that the effect of phytochemical is beneficial to health. To measure the association between the predicted effects of phytochemicals and ethnopharmacological evidence of plants, we calculated the semantic similarity between phenotype pairs on the Unified Medical Language System (UMLS) network. Moreover, we investigated the chemical properties of phytochemicals to confirm whether they are orally bio-available, drug available or effective on certain tissues. Finally, we inferred the health effects of 591 phytochemicals for 3832 phenotypes based on the integrated analysis of the molecular network, chemical properties and ethnopharmacological evidence. When we assessed the results, we found that our predictions cover many results which were reported in previous work. To conclude, the novelty of our method is threefold: (i) it is the first in silico method which identifies systemic health effects of phytochemicals by analyzing molecular properties, chemical properties and ethnopharmacological evidence; (ii) the large-scale analysis can be performed based on the integrated and structured molecular and phenotype information; and (iii) it can be used as a preliminary tool to screen medicinal agents from numerous phytochemicals.

## 2. Materials and Methods

### 2.1. Materials

Information about phytochemicals and the chemical composition of plants was collected from KTKP [22], TCMID [23] and FooDB [24]. Plants and their ethnopharmacological use were collected from KTKP, TCMID and Kampo [25]. Molecular targets of phytochemicals were collected from the DrugBank, the Drug Combination Database (DCDB) [26], the Comparative Toxicogenomics Database (CTD) [27], MATADOR [28], STITCH [29] and TTD [30] databases, and gene-phenotype associations were collected from the CTD database. A protein-protein interaction (PPI) network, including 19,093 nodes and 270,970 edges, was obtained from BioGrid version 3.4.136 [31] and the Context-Oriented Directed Associations (CODA) system [32]. A phenotypic network was collected from UMLS in the 2017AA version [33]. UMLS provides integrated information of various terminologies pertaining to biomedicine. The Metathesaurus is the main component of the UMLS, which is organized by biomedical concepts where each distinct concept is assigned to a concept unique identifier (CUI). We collected CUIs with broader (RB), narrower (RN) and other-related (RO) relationships among 11 types of UMLS relationships from related concept lists (File=MRREL.RRF), resulting in total 220,104 CUIs and 663,018 relationships.

For a gold-standard set, phytochemical derived drugs were collected from DrugBank version 4.3 [34]. Drug-phenotype associations were collected from DrugBank, CTD, ClinicalTrials.gov [35] and DCDB databases by exploiting the MetaMap tool to extract phenotype-related terms [36]. With inputs such as narrative text, MetaMap returns a ranked list of Metathesaurus concepts associated with each word of the input text. Among the Metathesaurus concepts categorized in semantic types, we used Metathesaurus concepts assigned to 20 semantic types out of 135 semantic types, which have related phenotypes such as “Disease or syndrome”, “Sign or symptom” and “Clinical attribute” (Table 1).

### 2.2. Method Overview

**Figure 1:**
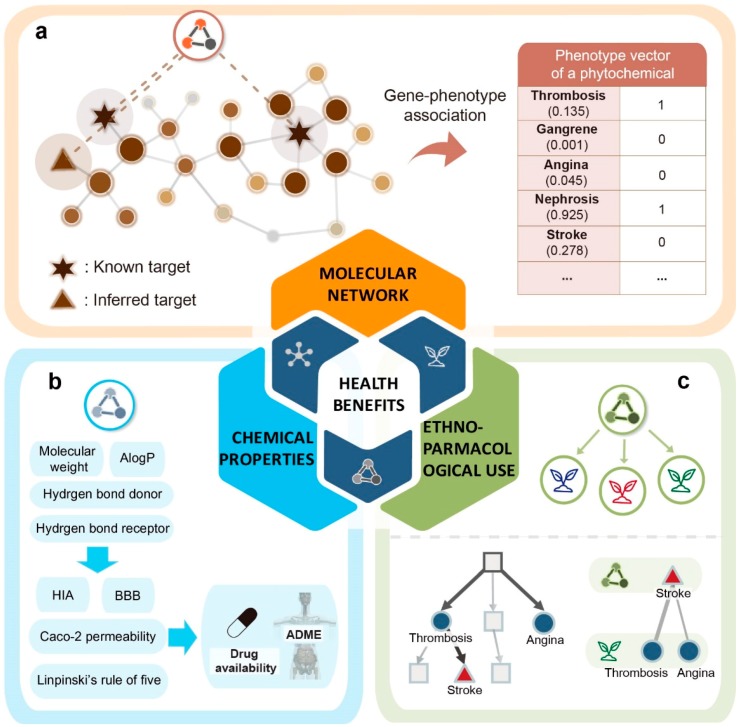
A systematic pipeline for the prediction of the health effects of phytochemicals. (**a**) Phenotype values of a phytochemical were obtained by calculating the propagated effects on the molecular network. In the molecular network, the random walk with restart (RWR) algorithm was performed based on direct targets (star) and indirect targets (triangle) of a phytochemical, in which the RWR results are shown as colored nodes. Based on gene-phenotype associations, sums of gene values are mapped to phenotypes. (**b**) For all phytochemicals, chemical properties, including physicochemical properties and physiological effects, were calculated. (**c**) Plants containing the phytochemical were extracted. For each extracted plant, we calculated the semantic similarity between the predicted health effect of the phytochemical and the ethnopharmacological effects of the plant. To do this, we constructed phenotypic network and calculated the shortest path length between phenotype pairs and depth of the phenotypes. Plants with the similarity score larger than the user-defined threshold were selected.

We designed a systematic pipeline to predict the potential health benefits of phytochemicals based on molecular and chemical properties of the phytochemicals, and ethnopharmacological evidence of the plants that contain the phytochemicals. For a query phytochemical, the algorithm works in three steps (Figure 1): (i) Inferring the systemic health effects of phytochemicals by calculating propagated effects on the molecular network and filtering statistically significant phenotypes; (ii) investigating bioavailability based on the physicochemical properties and physiological effects; and (iii) finding ethnopharmacological evidence.

In our previous study, we found that the phenotypic effects of drugs can be identified by calculating propagated drug effects on the molecular network [21]. We applied this method to phytochemicals to infer their systemic health effects. For this, we constructed phenotype vectors of phytochemicals (PVPs). Each vector contains health effects or disease-related terms for 3832 phenotypes defined by Medical Subject Headings (MeSH) and Online Mendelian Inheritance in Man (OMIM). The PVPs were generated by the following three steps. In the first step, propagated phytochemical effects were calculated by using the random walk with restart (RWR) algorithm on the molecular network (Figure 1a). The effects of phytochemicals are not limited to direct targets, but they are further propagated to interacting proteins. Therefore, initial values of the molecular network were assigned to the known and inferred targets of the phytochemicals, and the propagated effects of phytochemicals were calculated by applying the RWR algorithm. Consequently, this approach addresses the problem of the relatively small number of known targets of phytochemicals, compared to synthetic drugs. In the second step, phenotype values were calculated by combining propagated phytochemical effects based on gene-phenotype associations. Accordingly, phenotypes have high values when a drug directly binds to phenotype-associated genes or when drug targets are closely located to phenotype-associated genes. In the third step, PVPs were constructed by filtering statistically significant phenotypes from the inferred list of phenotypes, which were calculated from the second step. We then calculated the chemical properties, including physicochemical properties and physiological effects, to predict the bioavailability of phytochemicals (Figure 1b). Based on this result, we found phytochemicals which can be orally absorbed or can be delivered to certain tissues. Finally, ethnopharmacological evidence of phytochemicals was investigated (Figure 1c). For a query phytochemical, we first found plants containing the phytochemical. We then calculated the semantic similarity between the predicted health effects of the phytochemical and the ethnopharmacological use of the plants. If the semantic similarity score is significantly high, then we determine that the ethnopharmacological use of the plant is highly associated with the phytochemical. This process is meaningful since it implies that ethnopharmacological evidence can help to select more relevant results. Also, it can be used as an additional filtering criterion. We provide all predicted health effects, chemical properties and ethnopharmacological use evidence of the phytochemicals (Supplementary Data 1 and Data 2).

### 2.3. Inferring Health Effects of Phytochemicals on the Molecular Network

We constructed a molecular network based on PPI information and performed the RWR algorithm to investigate the propagated effects of phytochemicals. RWR simulates the random walker from its seed nodes and iteratively transmits the node values to the neighbor nodes, with the probabilities proportional to the corresponding edge weights [37,38]. To apply the RWR algorithm, we assigned initial values to seed nodes in the molecular network based on the target information of the phytochemicals. Target information of phytochemicals can be divided into two groups: direct and indirect associations. The direct associations contain binding information between phytochemicals and target proteins, while the indirect associations involve interactions caused by the changes in the expression of a protein, compound-induced phosphorylation, or influences of active metabolites of the phytochemicals. Information from both types of associations had to be taken into consideration, since the biological activity of a phytochemical can be changed from complex interactions within the molecular network, and the binding target information of phytochemicals is largely hidden compared to that of synthetic drugs. The initial values of a direct and indirect association were assigned as 1 and 0.3, respectively [21,27]. Second, the transition probability from a node to the neighbor node was calculated. We assumed that the transition probability represents the propagated drug effects on the molecular network. The transition probability vector of each node at time step *t* + 1 is defined as following equation:

$$p_{\left(t+1\right)}=(1-r)W^{T}p_{t}+rp_{0}$$

where *r* represents the restarting probability of the random walker at each time step, set to 0.7 in this study [38,39,40,41]. *W* represents the normalized adjacency matrix of the molecular network, *p_t_* is the probability vector of each node at time step *t*, and *p*_0_ represents the initial probability vector. The RWR algorithm simulates the random walker until all nodes reach the steady state (*p_t_*_+1_ − *p_t_*< 10^−8^). We then mapped the RWR results to phenotypes based on the gene-phenotype associations. In this step, we found all genes which are therapeutic targets or biomarkers of certain phenotypes and mapped the sum of these gene values, which were obtained from RWR results, to the corresponding phenotypes. Through this process, we obtained a list of phenotype values for each phytochemical.

These phenotype values calculated from propagated effects do not necessarily represent the extent of the relationship between a phytochemical and phenotype. Even if a phenotype value calculated from propagated effects is high, it may not mean that the phytochemical is highly related to the phenotype. In cases where there are many phenotype-associated genes, or when there is a large number of target proteins for a phytochemical, overall phenotype values increase stochastically. To overcome this problem, we generated random PVPs and compared them to a list of inferred phenotype values to select phenotypes with significant values. A random PVP was generated by randomly selecting targets of a phytochemical from a fixed number of target proteins. For each phytochemical, 1000 random PVPs were generated, and phenotypes with an empirical *p*-value lower than 0.01 were selected. The *p*-value was calculated from the following equation:

p=(r+1)/(n+1)

where *n* is the number of random PVPs and *r* is the number of PVP values that are larger than the phenotype value [42]. The raw values of PVPs were then replaced with binary values, where only those with *p*-values lower than 0.01 are given the value one. From this process, PVPs, which consist of binary values, were generated with filtered statistically significant phenotypes from a large number of inferred candidates of phytochemical effects.

### 2.4. Calculating Chemical Properties of Phytochemicals

The chemical properties of phytochemicals were calculated to provide an understanding of the physicochemical properties and physiological effects (Figure 1b). Physicochemical properties include molecular weight, log of the octanol-water partition coefficient (AlogP), hydrogen-bond donors, hydrogen-bond acceptors, and rotatable bond count. Physiological effects include human intestinal absorption (HIA), Caco-2 permeability, blood-brain barrier (BBB) permeability and Lipinski’s rule of five (RO5). By utilizing the physiological effects of phytochemicals, we can predict various functional activities of the compounds on the human body. For example, we can predict the in vivo absorption of phytochemicals across the gut wall based on the Caco-2 permeability [43]. Phytochemicals are required to cross the BBB to have a neuroactive function. In this study, HIA and BBB values are calculated with Shen’s work [44], while Caco-2 permeability is calculated through the quantitative structure–activity relationship (QSAR) model [45]. RO5 and other physicochemical properties are calculated with the Chemistry Development Kit (CDK) Descriptor Calculator [46].

### 2.5. Finding the Ethnopharmacological Use of Phytochemicals

**Figure 2:**
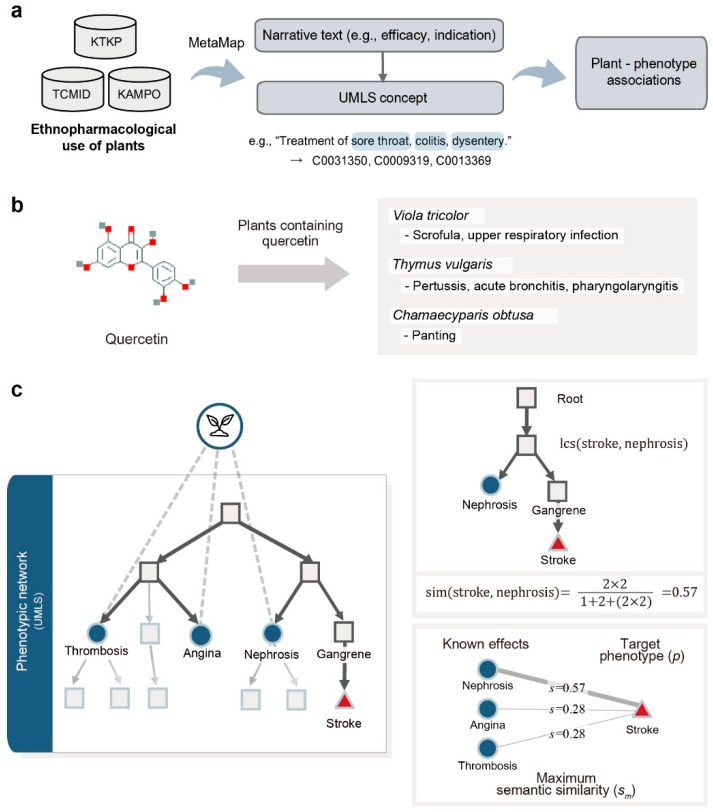
An overview of the findings of the ethnopharmacological use of phytochemicals. (**a**) From public databases, we collected ethnopharmacological evidence of medicinal plants. We then extracted phenotype-associated terms from the narrative text of the collected information by applying the MetaMap tool. (**b**) For a queried phytochemical, plants containing the phytochemical were extracted. (**c**) For each extracted plant, we mapped its ethnopharmacological effects to the phenotypic network (blue circle). Then, we calculated semantic similarities between all possible pairs of predicted health effects of phytochemicals and ethnopharmacological effects of the plant. In this example, the semantic similarity between stroke and nephrosis is 0.57, based on the semantic similarity formula, because the depth of *lcs* is 2, the shortest path length between nephrosis and *lcs* is 1 and the shortest path length between stroke and *lcs* is 2. Plants with a similarity score larger than 0.8 were selected.

We investigated the ethnopharmacological use of plants to provide further evidence of the predicted health effects of phytochemicals. The ethnopharmacological information, such as efficacy or indications collected in articles from scientific journals and documents of traditional medicine, is generally described in narrative text. Moreover, there are complex associations between phenotypes, such as synonyms and symptoms of diseases. Therefore, it is difficult to determine whether certain ethnopharmacological evidence is associated with the phenotype of interest. To extract plants which have ethnopharmacological evidence of the predicted effects of a phytochemical, (i) phenotype terms should be extracted and structuralized from the narrative text, and (ii) the complex relationship between phenotypes should be quantified. To solve this problem, we first extracted phenotype-associated terms from the narrative text by applying the MetaMap tool (Figure 2a). Next, plants containing the queried phytochemical were found based on external database information (Figure 2b). Next, the phenotypic network was constructed based on the hierarchical relationship of UMLS [33], and the semantic similarities between phenotypes were calculated (Figure 2c). A relationship between two general phenotype concepts, such as neoplasms and cardiovascular diseases, would result in a reasonably large difference, while one between two closely related concepts such as coronary stenosis and coronary vasospasm would result in a small difference. Semantic similarity can measure the quantitative relatedness between phenotypes by considering the distance and depth of phenotypes in the network. We applied the semantic similarity measure proposed by Wu & Palmer (wup) and defined as [47]:

$$\mathrm{sim}(c_{1},c_{2})=\frac{2\times \mathrm{depth}(lcs(c_{1},c_{2}))}{\mathrm{path}(c_{1},lcs(c_{1},c_{2}))+\mathrm{path}(c_{2},lcs(c_{1},c_{2}))+2\times \mathrm{depth}(lcs(c_{1},c_{2}))}$$

where *lcs* (*c*_1_,*c*_2_) is the lowest common subsumer of concepts *c*_1_ and *c*_2_. Based on this method, we can calculate the distance between the inferred effect of a phytochemical and the ethnopharmacological use of a plant. In this study, we assumed that the phenotype pair is highly associated when a semantic similarity score is larger than 0.8. Therefore, we calculated semantic similarities between all possible pairs of predicted health effects of a phytochemical and ethnopharmacological effects of the plant, and plants with the similarity score larger than 0.8 were selected. We showed that the effect type of predicted health effects is likely to be beneficial by investigating the evidence of the ethnopharmacological use of plants based on semantic similarity. 

## 3. Results

### 3.1. Inferred Health Effects of Phytochemicals

**Figure 3:**
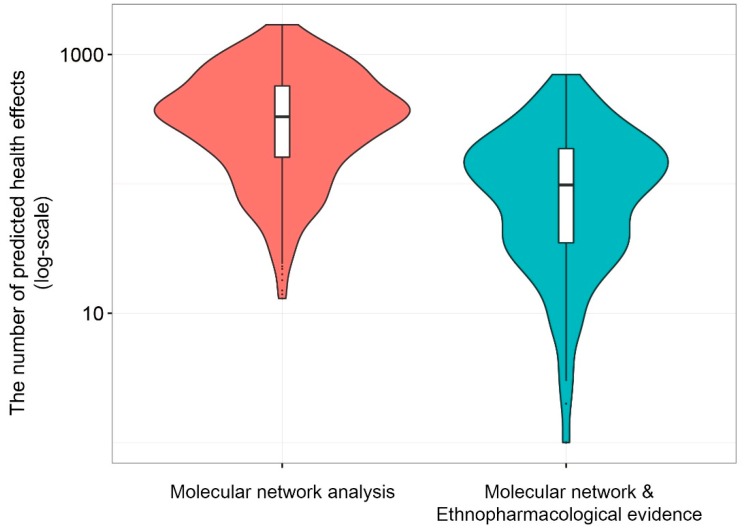
The distribution of the number of predicted health effects. The distribution of the number of predicted health effects by molecular network analysis (red violin plot). The mean of predicted health effects is 415.6 ± 27.3. Next, we investigated the intersection between predicted health effects of the phytochemicals and ethnopharmacological use of the plant containing the phytochemicals. The distribution of the number of predicted health effects by molecular network analysis and ethnopharmacological use evidence (blue violin plot). The mean of predicted health effects is 129.1 ± 11.4.

From public databases, we were able to collect information for 2136 phytochemicals found in 1212 plants. However, the information on chemical structures was only available for 512 of the phytochemicals (23.9%), while the molecular target was known for only 591 of them (27.6%). Hence, we predicted the potential health effects of 591 phytochemicals by investigating their propagated effects on the molecular network based on molecular target information and mapping the effects to phenotypes. From the results, an average of 415.6 ± 27.3 (confidence interval = 0.95) health effects were predicted for each phytochemical (Figure 3). Since there are many candidate health effects in the molecular network analysis, and their detailed effect types are unknown, we further investigated the intersection between the predicted health effects of the phytochemicals and the ethnopharmacological use of the plant containing the phytochemicals. The results indicated that 31% of the predicted health effects had ethnopharmacological evidence (129.1 out of 415.6 health effects).

Next, the physiological effects of phytochemicals were confirmed (Table 2). To do this, we investigated RO5, HIA, Caco-2 permeability and BBB permeability for 512 phytochemicals (Supplementary Data 2). For example, 446 phytochemicals were found to satisfy RO5. Additionally, 401 phytochemicals were confirmed to satisfy both RO5 and HIA.

### 3.2. Performance Evaluation

Our method predicts the potential health effects of phytochemicals from the integrated analysis (Supplementary Data 1). The core information used in such a prediction is the propagated effects of phytochemicals obtained from the molecular network. Therefore, we evaluated the performance of the prediction by calculating precision (*p*) and recall (*r*) values [48]. To do this, we collected the experimentally validated information as the gold-standard positive set. Indications from DrugBank were used as the set for therapeutic effects, while information from Side Effect Resource (SIDER) was used as that for side effects. Furthermore, considering that information on phytochemicals is limited for DrugBank and SIDER, we additionally collected potential candidate effects from CTD as the silver standard positive set to address a large number of phytochemicals.

In the prediction of phytochemical effects, large class skew and large changes in class distributions are common, because the negative set is not available. Therefore, many studies have excluded gold-standard positive sets from all possible health effects and used the remaining as the gold-standard negative set [49,50,51]. To see the effect due to class skew, we calculated precisions for different positive/negative ratios to evaluate the precision performance in the various skewness of datasets (Table 3) [52]. To do this, we generated a negative set by random sampling without replacement of the phytochemical and phenotype associations in different ratios. In each ratio, the negative set was generated ten times, and the performance for each case was evaluated by averaging the results. Moreover, since we predict an average of 415.6 potential health effects per phytochemical, the precision is very low (*p* = 0.006 ± 0.001 and 0.049 ± 0.010, respectively). This is natural, because the correct answer in DrugBank or SIDER is only a fraction of all health effects of phytochemicals. Therefore, we evaluated the precision performance by adjusting skewness between the positive set and negative set, and we confirmed that molecular network analysis predicts health effects with high precision. Next, we checked the recall performance. Out of 270 therapeutic effects of 61 phytochemicals, our method covered 191 phenotypes (*r* = 0.738 ± 0.062). Similarly, for side effect prediction, our method covered 1059 phenotypes among the total 1784 phenotypes of 60 phytochemicals (*r* = 0.576 ± 0.061). In potential candidate effect prediction, our method covered 119,233 phenotypes among the total 136,862 phenotypes of 453 phytochemicals (*r* = 0.909 ± 0.011). Overall, the prediction of health effects with molecular network analysis shows a good performance.

Next, we compared the prediction performance with and without considering ethnopharmacological evidence (Table 4). To consider the ethnopharmacological evidence, predicted health effects were filtered based on the presence of the ethnopharmacological use of the plant containing the phytochemical. The results show that the precision performance is increased when the ethnopharmacological evidence is considered, in terms of predicting therapeutic (*p* = 0.014 ± 0.003) and potential candidate effects (*p* = 0.563 ± 0.059). Interestingly, we found that the precision value of the side effects prediction was reduced because we only used the therapeutic use case information in the ethnopharmacological evidence. This indicates that the ethnopharmacological evidence helps distinguish between types of phytochemicals effects, such as therapeutic or side effects, which is one of the disadvantages of molecular network analysis. 

Lastly, we confirmed the performance improvement using chemical properties. Because phytochemicals must pass through the BBB to regulate a neuroactive function, we compared the performance of the prediction of neurological disorder for the two independent sets by selecting phytochemicals based on BBB permeability. From the results, we found that the precision and recall values of the set that crosses the BBB (*p* = 0.611 ± 0.046, *r* = 0.725 ± 0.033) are much higher than the set that does not cross the BBB (*p* = 0.312 ± 0.052, *r* = 0.558 ± 0.042). Overall, our results indicate that the integrated analysis can predict health benefits of phytochemicals more accurately than analysis using individual information.

### 3.3. External Literature Validation

In this section, we aimed to provide additional performance evaluations using the external data set that has not been used in our prediction method. We evaluated the reliability of our prediction results that are phytochemical-phenotype associations by checking the frequency of co-occurrence of phytochemicals and phenotypes in the PubMed abstract. Our basic assumption here is that if our method predicts reliable associations, then those phytochemical-phenotype associations would have higher probability of co-occurrence in previous studies than random phytochemical-phenotype associations would. Therefore, we made two independent sets based on the predicted health effects of phytochemicals. First, the predicted association set was generated by selecting phytochemical-phenotype associations, which were predicted as positive by molecular analysis, oral-availability and ethnopharmacological evidence. Second, for the control, the random association set was generated by randomly sampling the same number of samples from phytochemical-phenotype associations, without the aforementioned predicted association set.

We used 13,200,786 PubMed abstracts that were published from 1950–2013 for external literature validation. For the predicted phytochemical-health effects, we counted co-occurrences of phytochemical-phenotype terms (*n_c_*) from PubMed abstracts, calculated the Jaccard index (JI), and conducted the Fisher’s exact test (*n_p_*) and false discovery rate (FDR) test (*n_q_*) (Table 5). We also performed the Mann-Whitney *U* test and calculated the corresponding *p*-values to check the statistical difference of the literature evidence between the predicted and random association sets [53]. A *p*-value of the Mann-Whitney *U* test lower than 0.05 was considered statistically significant. 

The co-occurrence value was calculated by counting the number of PubMed abstracts where a phytochemical and its corresponding phenotype were in a same sentence. The average number of co-occurrence of the predicted association set (*n_c_* = 1.25) were 13.8 times larger than that of the random association set (*n_c_* = 0.09). Also, we normalized the co-occurrence value by the Jaccard index to correct the differences in the frequency of phytochemicals and phenotypes [54]. To do this, we additionally calculated an occurrence value (*n_o_*) by counting the number of PubMed abstracts that contain both or either of a phytochemical or a phenotype. For each phytochemical-phenotype association, the Jaccard index was calculated by dividing *n_c_* by *n_o_*. For example, assume that we calculate the Jaccard Index for the phytochemical-phenotype pair “quercetin—stroke”. If there are 50 abstracts that mention both quercetin and stroke and there are 200 abstracts that mention either or both, then the Jaccard Index value for this pair would be 0.25. From the results, the average Jaccard index value of the predicted association set (JI = 1.8 × 10^−4^) was 18.9 times higher than that of the random association set (JI = 9.5 × 10^−6^). Furthermore, we performed Fisher’s exact test to find the significant associations (*p*-value < 0.001). To get the Fisher’s test value of each association, we counted the number of PubMed abstracts based on whether they included the phytochemical and target health effect. The results indicate that the number of significant associations of the predicted association set (*n_p_* = 2984) was 4.8 times higher than that of the random association set (*n_p_* = 612). However, when a large number of associations are evaluated, multiple testing problems arise and lead to many false positive results. Therefore, we additionally performed the FDR test and found associations satisfying a *q*-value lower than 0.05 [55]. The results indicate that the number of associations satisfying the *q*-value criteria from the predicted association set (*n_q_* = 1341) was 4.9 times higher than that from the random association set (*n_q_* = 274). In addition, the *p*-values of the Mann-Whitney *U* test indicated that the difference in the literature evidence among the predicted and random association sets was significant. These results showed that our method can be used as a tool to identify the health effects of phytochemicals.

### 3.4. Case Studies

To further illustrate the potential of this algorithm in finding phytochemicals with possible medicinal effects against specific diseases, we selected a few phytochemicals, such as choline, isoquercitrin and niacin, as case study subjects on whether there is evidence of their health benefits. To begin with, our method predicted that choline could be effective against 1151 phenotypes, such as hypertension, neurological diseases and hypoimmunity. Of these, 515 phenotypes were supported by ethnopharmacological evidence. From this list, the top 10 phenotypes with the most ethnopharmacological use evidence were selected. Then, we manually searched through the ClinicalTrials.gov database to check whether any clinical trials were performed regarding these phenotypes using choline. Interestingly, there were two phase four trials done with choline to treat neurological disorders (ethnopharmacological evidence *n_e_* = 48, rank = 2). The first trial was by Daewoong Pharmaceutical Co. LTD. in 2016, where the study was designed to test the efficacy of the choline alfoscerate on cognitive improvements of patients with cerebrovascular injury in Alzheimer’s disease. Along with this study, there are several animal studies [56,57] and another clinical trial that support the dietary supplement of choline for the possible prevention of dementia and memory loss [58]. The second trial related to neurological disorders was by Seoul National University Hospital in 2013, in which the study focused on the cognitive impairment of post-stroke patients treated with choline alfoscerate. Such an approach to cognitive damage with a choline supplement is also supported with various animal studies [59,60] and a clinical trial [61]. Although one may claim that the range of the term “neurological disorder” covers a wide spectrum of phenotypes, it is important to note that the specific phenotypes of the clinical trials mentioned above, which are Alzheimer’s disease (*n_e_* = 31, rank = 81) and cognitive impairment (*n_e_* = 3, rank = 419), were also on the list of possible target phenotypes of choline. Furthermore, there was also a phase four clinical trial for pain (*n_e_* = 44, rank = 6) by Columbia University in 2016, where the researchers planned to study the effects of taking choline to decrease postoperative pain. The anti-nociceptive effects of choline have been suggested with various in vivo animal studies as well [62,63].

The evidence that illustrates the effectiveness of the algorithm in finding the effects of phytochemicals on potential phenotypes can be also found in the other two phytochemicals—isoquercitrin and niacin—as well. The results of these case studies are organized, along with those of the choline, in Table 6. In each case it can be seen that, for the phenotypes that are found to have a high potential relationship to the phytochemical, there have been related clinical trials, many of which are already on phase three or four. As the algorithm discussed in this paper agrees with the conclusions from the aforementioned clinical trials and experiments, it is possible to clearly see that the algorithm can provide productive and plausible insights into potential therapeutic relationships between phytochemicals and diseases of interest. 

## 4. Discussion

In this study, we introduced an integrated analysis to predict the health benefits of phytochemicals. By investigating the propagated effects of 591 phytochemicals in the molecular network, we inferred potential health effects of those phytochemicals for 3832 phenotypes. For all phytochemicals, we investigated various physicochemical and physiological properties, such as HIA, Caco-2 permeability, BBB permeability and RO5, so that the results can be used in further studies, such as on oral bioavailability, drug availability and tissue specificity. Moreover, we provided evidence on the ethnopharmacological use of plants to support the predicted health effects. Herbal medicine has accumulated information on medicinal plants for thousands of years. Recent studies have demonstrated that herbal medicine information can be used as an important resource in drug or functional food development [64,65,66]. Therefore, we supported our results by investigating whether the predicted health effects of phytochemicals are also found in the ethnopharmacological use of plants containing the phytochemicals. For example, when we are looking for effective phytochemicals against neurological disorders, we first check the 3832 predicted health effects of phytochemicals which were inferred from molecular network analysis. Then, we select phytochemicals with positive physiological effects for RO5 and BBB permeability. Finally, we can find phytochemical candidates for neurological disorders by investigating whether the phytochemicals have ethnopharmacological evidence for neurological disorders. Performance evaluation revealed that the accuracy of predictions using all three types of information together was better than that using each individual type. Such improvement can be attributed to how each type of information fills in each other’s gap in content. Mere health effect candidates can be obtained with simple molecular network analysis, but the results would have been based on information with two major gaps for proper pharmacological studies: tissue specificity and effect type prediction. By considering the phytochemical’s physiological properties in the algorithm, it became possible to consider tissue specificity, thereby improving the overall prediction. Likewise, utilizing ethnopharmacological evidences allowed to overcome a major drawback of molecular network analysis, which is that the prediction does not consider effect types. The effect types, such as therapeutic or side effects from experience, help to narrow down possible medicinal influences of the phytochemicals on human body.

The strength of this algorithm is further highlighted by illustrating that there are several clinical trials already deep into phases three or four that are investigating the potential effects of the selected phytochemicals on their predicted phenotypes. This implies that the algorithm can be utilized to effectively predict the potential targets of the phytochemicals and vice versa. Also, this shows the application potential of the proposed method. Thus, productivity in such studies of medicinal effects can be expected to improve.

There are additional considerations that may improve our method. First, although this study utilized the ethnopharmacological use of the plants as important information to analyze the effects of phytochemicals, we did not consider the combination effects of phytochemicals. Because plants are composed of many phytochemicals, pharmacological effects of plants are often caused by the combined actions of multiple phytochemicals, as well as the individual actions of phytochemicals. However, this issue is very complex since the number of candidate combinations has increased exponentially with the increase of the number of considered phytochemicals. Second, the dosage of phytochemicals is not taken into an account in the method, although the health effects can be varied by different amount of chemicals taken. Until now, most studies have focused on the dose-response relationship for drugs, whereas only a few computational methods have calculated the expected content-response relationship for phytochemicals [67,68]. Lastly, current knowledge of phytochemicals is limited, and hence only a small proportion of phytochemicals could be analyzed [69]. In this study, we only consider 591 phytochemicals, since the information on chemical structure and molecular targets of phytochemicals are mostly hidden. Nevertheless, these limitations can be taken into an account for further experiments or improved computational methods. With these further improvements, our method can be used as an in silico screening tool to provide a list of health effects of phytochemicals in a cost-effective manner.

## 5. Conclusions

This study identified the health benefits of phytochemicals by utilizing various phytochemical properties, including molecular and chemical properties, along with ethnopharmacological evidence. Based on the known and inferred effects from gold and silver standard datasets, we confirmed that the health effects of phytochemicals could be successfully predicted with high coverage. We believe that the identification of the potential health benefits of phytochemicals may be a key factor to provide further insights into the discovery of drugs or functional foods.

## Figures and Tables

**Table 1:** Health effects-related UMLS semantic types. Among 135 semantic types, the following 20 semantic types were selected as related to health effects.

Abbreviation	Semantic Type
acab	Acquired Abnormality
anab	Anatomical Abnormality
biof	Biologic Function
cgab	Congenital Abnormality
comd	Cell or Molecular Dysfunction
dsyn	Disease or Syndrome
emod	Experimental Model of Disease
fndg	Finding
inpo	Injury or Poisoning
lbtr	Laboratory or Test Result
menp	Mental Process
mobd	Mental or Behavioral Dysfunction
neop	Neoplastic Process
patf	Pathologic Function
phsf	Physiologic Function
sosy	Sign or Symptom
clna	Clinical attribute
hops	Hazardous or Poisonous Substance
bpoc	Body Part, Organ, or Organ Component
tisu	Tissue

**Table 2:** The number of phytochemicals which satisfy RO5, HIA, Caco-2 and BBB. We also investigated the number of phytochemicals which satisfy two physiological effects.

	RO5	HIA	Caco-2	BBB
RO5	446	401	280	365
HIA		482	330	407
Caco-2			335	303
BBB				428

**Table 3:** Precision and recall performance of molecular network analysis in predicting therapeutic effects, side effects and potential candidate effects.

	Skewness	Therapeutic Effects	Side Effects	Inferred Candidates
Precision	1:1	0.921 ± 0.032	0.922 ± 0.021	0.942 ± 0.005
	1:10	0.518 ± 0.059	0.432 ± 0.040	0.706 ± 0.013
	All	0.006 ± 0.001	0.049 ± 0.010	0.522 ± 0.022
Recall	All	0.738 ± 0.062	0.576 ± 0.061	0.909 ± 0.011

**Table 4:** Precision performance of the method, which uses molecular network analysis and ethnopharmacological use evidence in predicting therapeutic effects, side effects and potential candidate effects.

Skewness	Therapeutic Effects	Side Effects	Inferred Candidates
1:1	0.941 ± 0.035	0.761 ± 0.033	0.948 ± 0.014
1:10	0.541 ± 0.069	0.319 ± 0.055	0.732 ± 0.037
All	0.014 ± 0.003	0.025 ± 0.005	0.563 ± 0.059

**Table 5:** Literature validation was performed by comparing co-occurrence, the Jaccard index and Fisher’s exact test values between predicted and random association sets. Statistical significance was calculated by the *p*-value of the Mann-Whitney *U* test.

	Co-Occurrence	Jaccard Index	*p*-Value ^1^	*q*-Value ^2^
Predicted association set	1.25	1.8 × 10^−4^	2984	1341
Random association set	0.09	9.5 × 10^−6^	612	274
Mann-Whitney *U* test, *p*-value	<0.001	<0.001	<0.001	<0.001

^1^ The number of phytochemical-health effects associations which satisfy the *p*-value of Fisher’s exact test is lower than 0.001. ^2^ The number of phytochemical-health effects associations which satisfy *q*-value of FDR test is lower than 0.05.

**Table 6:** Summary of evidence indicating potential health effects of exemplary phytochemicals: isoquercitrin, niacin and choline.

Phytochemical	Potential Health Effects	Rank	*n_e_*	Clinical Trials (Phase)	Exemplary Studies (PMID)
Choline	Neurological disorder	2	48	-	-
	Alzheimer’s disease	81	31	NCT02648906 (Phase 4)	12787861, 15647594 12637119, 7913981
	Cognitive impairment	419	3	NCT01363648 (Phase 4)	21195433, 19304299 26366063, 23403474
	Pain	6	44	NCT00720343 (Phase 4)	15780465, 19372354, 16942753, 29082318
Isoquercitrin	Hypertension	2	64	NCT01691404 (Not Applicable)	20134098, 25460361 17951477, 16636461
	Thrombosis	10	56	NCT02195232 (Phase 2, 3)	12854360, 15234778 20148891, 20626032
Niacin	Neurological Disease	2	128	-	-
	Parkinson’s Disease	59	65	NCT03462680 (Not Applicable)	26273459, 25455298 26988916, 18381761
	Heart condition	7	117	NCT00120289 (Phase 3) NCT00000599 (Phase 3)	28057839, 23916935 28927896, 10924076
	Vascular disease	9	113	-	-
	Cardiovascular disease	20	105	NCT00715273 (Phase 4) NCT02003638 (Not Applicable)	3295315, 19159436 15258194, 24641964

## Supplementary Materials

The following are available online at http://www.mdpi.com/2072-6643/10/8/1042/s1, Supplementary Data 1: Predicted health effects of phytochemicals, Supplementary Data 2: Chemical properties of phytochemicals.
